# Rehabilitation of Upper Limb Motor Impairment in Stroke: A Narrative Review on the Prevalence, Risk Factors, and Economic Statistics of Stroke and State of the Art Therapies

**DOI:** 10.3390/healthcare10020190

**Published:** 2022-01-19

**Authors:** Saba Anwer, Asim Waris, Syed Omer Gilani, Javaid Iqbal, Nusratnaaz Shaikh, Amit N. Pujari, Imran Khan Niazi

**Affiliations:** 1 School of Mechanical & Manufacturing Engineering, National University of Sciences and Technology (NUST), Islamabad 45200, Pakistan; sanwer.bmes19smme@student.nust.edu.pk (S.A.); asim.waris@smme.nust.edu.pk (A.W.); omer@smme.nust.edu.pk (S.O.G.); j.iqbal@ceme.nust.edu.pk (J.I.); 2 Faculty of Health & Environmental Sciences, Health & Rehabilitation Research Institute, AUT University, Auckland 0627, New Zealand; nusrat.shaikh@aut.ac.nz; 3 School of Physics, Engineering and Computer Science, University of Hertfordshire, Hatfield AL10 9AB, UK; amit.pujari@ieee.org; 4 School of Engineering, University of Aberdeen, Aberdeen AB24 3FX, UK; 5 Center of Chiropractic Research, New Zealand College of Chiropractic, Auckland 1060, New Zealand; 6 Center for Sensory-Motor Interaction, Department of Health Science & Technology, Aalborg University, 9000 Alborg, Denmark

**Keywords:** stroke, rehabilitation, tele rehabilitation, electric stimulation, virtual reality (VR), epidural, NIBS, risk factors, modifiable, non-modifiable

## Abstract

Stroke has been one of the leading causes of disability worldwide and is still a social health issue. Keeping in view the importance of physical rehabilitation of stroke patients, an analytical review has been compiled in which different therapies have been reviewed for their effectiveness, such as functional electric stimulation (FES), noninvasive brain stimulation (NIBS) including transcranial direct current stimulation (t-DCS) and transcranial magnetic stimulation (t-MS), invasive epidural cortical stimulation, virtual reality (VR) rehabilitation, task-oriented therapy, robot-assisted training, tele rehabilitation, and cerebral plasticity for the rehabilitation of upper extremity motor impairment. New therapeutic rehabilitation techniques are also being investigated, such as VR. This literature review mainly focuses on the randomized controlled studies, reviews, and statistical meta-analyses associated with motor rehabilitation after stroke. Moreover, with the increasing prevalence rate and the adverse socio-economic consequences of stroke, a statistical analysis covering its economic factors such as treatment, medication and post-stroke care services, and risk factors (modifiable and non-modifiable) have also been discussed. This review suggests that if the prevalence rate of the disease remains persistent, a considerable increase in the stroke population is expected by 2025, causing a substantial economic burden on society, as the survival rate of stroke is high compared to other diseases. Compared to all the other therapies, VR has now emerged as the modern approach towards rehabilitation motor activity of impaired limbs. A range of randomized controlled studies and experimental trials were reviewed to analyse the effectiveness of VR as a rehabilitative treatment with considerable satisfactory results. However, more clinical controlled trials are required to establish a strong evidence base for VR to be widely accepted as a preferred rehabilitation therapy for stroke.

## 1. Introduction

Stroke is categorized as one of the most concerning global health issues as it is a serious and common disabling factor worldwide. Recent figures suggest that around 650 million people are of 60 years or above age globally, and this number is expected to increase up to 2 billion by 2050 [1]. Ageing and urbanization are two powerful drivers of stroke. The elderly population is at higher risk of experiencing a stroke, but stroke can be prevented to some extent by dealing with the modifiable menace factors such as physical inactivity, drugs, unhealthy diet, and tobacco so that problems such as hypertension, high blood pressure, and diabetes, which are the root causes of the epidemic, may be managed [2]. A recent report by the World Health Organization (WHO) [3] states that stroke is the primary cause of disability and deaths among the senior population after heart disease. In 2012, almost 7 million (11.1%) people worldwide died due to stroke. Through stroke prevalence and incidence data [4] collected from fourteen EU and six EFTA countries, based on observing the lifestyle of the urban population, a remarkable increase in stroke numbers by 2025 is predicted. Post-stroke care programs impose a substantial economic burden on society. Hence, it is crucial to understand the significant cost drives (incurred in stroke rehab) and fill the information aperture to help compose effective public health care and rehabilitation policy. There is a considerable survival rate of stroke patients causing long term health consequences for the patients and their families [4]. It is expected that the prevalence of stroke burden will increase in the next 2–3 decades. Recent years have seen noticeable improvements in the management of stroke rehabilitation [5]. However, significant and consistent improvements are still needed to meet the predicted rise in the number of stroke survivors and improve care quality. Modern developments in the medical world are playing a pivotal role in rehabilitating and managing the stroke population and related economic burden [5]. Further, the promise of technological developments has renewed the interest of researchers and clinical experts in rehabilitation interventions for post-stroke treatments.

### Method

In this review, we have focused on the importance of stroke rehabilitation while explaining the socio-economic burden of this disease and related risk factors narratively. Considering the increasing popularity and evidence of the benefits of technology-aided rehabilitation approaches, some commonly used stroke therapies to regain muscle activity have been reviewed. The presented review has been divided into two sections. The first section appraises the stroke statistics describing the prevalence rate, economic cost (treatment and home-care services) and related risk factors (modifiable and non-modifiable). In the second section, commonly used technology-enabled rehabilitation stroke-therapies have been reviewed. Randomized controlled trials and experimental studies have also been included to give the reader an accurate and clear idea about the disease factors and treatment therapies. Relevant randomized control studies, meta-analyses, and literature reviews were identified from Google Scholar. To search the articles related to upper limb rehabilitation, specifically using different therapeutic, robotic, stimulative (via surface and implanted electrodes), and VR approaches, the following combinations of the keywords were used:

- Functional electric stimulation (FES), neuromuscular electrical stimulation (NMES), transcranial direct current stimulation (TDCS) and transcranial magnetic stimulation (TMS) and electric stimulation.
- Upper limb, arm, and upper extremity.
- Robotic, orthosis, exoskeleton, assistive device, arm support, and tele rehabilitation.
- Muscle activation, epidural, and cortical stimulation.
- Sensory peripheral stimulation, and motor skills.
- Upper extremity immobilization and cerebral plasticity model.
- Physical therapy, VR therapy, and virtual rehabilitation.

Relevant publications in referenced papers have also been considered for composing an authentic and comprehensive review of the literature. Articles included in this review are based on the following criteria:

- Included rehabilitation therapies are implementable for the upper limb.
- Included therapeutic interventions are applicable for acute and chronic stroke patients.

## 2. Stroke Statistics

Considering the prevalence statistics of stroke and its disastrous impact on the economy associated with the treatment, medication, and post-stroke care, it is crucial to take some cost-effective and rehabilitative measures and fill the information gap. In a statistical review [5] that included 42 studies about economic aspects of post-stroke care (PSC) and treatment, it was concluded that PSC cost was higher in America (4850 USD/month) and minimum in Australia (752 USD/month) (see Table 1). In this study, data were collected from MEDLINE, Scopus, Google Scholar, and Cochrane results from 2000–2016 and results were extracted systematically for post-stroke care (PSC) services. Finally, the economic figures were converted to the 2015 USD, then the total PSC cost per month for a patient was calculated. This nursing care and rehabilitative therapies were the primary cost contributors [5]. In Europe, almost 1 million stroke cases are reported each year, and the number of registered stroke survivors is 6 million. This population requires an efficient health care system and a significant economic investment privately and publicly by the government [6]. Briefly, in 27 EU countries, the annual estimated cost for PSC and treatment is 27 billion euros (8.5 billion indirect and 18.5 billion direct medical expense) [7]. In 2008, the total stroke treatment cost in the USA was 65.5 billion USD (indirect cost 33% and direct cost 67%). The American Heart and Stroke Association has estimated the total stroke care cost to be 184.1 billion USD by 2030 [8]. Depending upon the severity and consequences of stroke, a patient may require lifetime care. Considering this, the financial and clinical cost of the epidemic is of direct relevance to the public healthcare system. The average cost for stroke rehabilitation and medication services in the United States in the years 2001–2005 was USD 11,145 per patient after being discharged, in which USD 7318 were spent on rehabilitation and USD 3376 on medication [8,9,10]. These numeric figures indicate the significance of acquiring advanced, cost-effective, and user-friendly rehabilitation technologies to meet the demand of the expected stroke population for at least the next 2–3 decades.

Another study analysed data from numerous registries and research databases; specifically, the expenses for acute and post-acute stroke management were calculated, including the direct health care and municipal services and indirect productivity losses using Modified Rankin Scale (mRs) per stroke category and functional disability [11]. Based on the stroke statistics and relevant facts presented in Table 2 and Table 3. it can be concluded that working on the rehabilitation of stroke patients and improving their functional ability is necessary for themselves and their families, as well as to lessen the economic burden on the society (see Table 4).

### 2.1. Stroke Risk Factor

Being a heterogeneous disease, stroke has contrasting consequences on human physiology, depending on whether the stroke is subarachnoid haemorrhage (SAH), ischemic stroke. or intraparenchymal haemorrhage (IPH), each varying in pathophysiology [11]. Epidemiologic studies can help identify the risk factors that lead to stroke. Marking the risk factors for certain precarious diseases help deal with the morbidity (see Table 2).

#### 2.1.1. Non-Modifiable Risk Factors

- Age

Stroke incidences increase with age between 45–85 years and almost double with each passing decade. The risk of having a stroke is highest among 55–65 years. In a study of stroke prevalence in the UK, it has been revealed that at the age of 40 there are 10 deaths/100,000 population, whereas there are 100 deaths/100,000 population at the age of 75. The stroke rate is doubled in both genders after 55 for each passing decade. Age is declared as an independent unmodifiable risk factor for experiencing intracranial atherosclerosis (ICAD), and its prevalence increases with each passing decade (23% for age group 50–60 years, 43% for 60–70 years, 65% for 70–80 years, and 80% for the population >80 years) [12].

- Heredity/Family stroke history

Heredity is one of the well-studied risk factors. Previous studies have divulged that genetic factors play an endeavouring role in developing premature ICAD-atherosclerosis in the body’s vascular system via proliferation of smooth muscle, angiogenesis impairment, and endothelial injury [13,14].

- Ethnicity

Results of a stroke study to determine the ethnicity as a risk factor showed that black people are at 2.1 times greater risk of subarachnoid haemorrhage and 1.4 times greater risk of intracerebral haemorrhage than white people [15]. The ratio of affected population with intracranial stenosis is higher in Hispanic and African Americans than white Americans. A comparative autopsy supports this data study (aorta and coronary arteries) for ICAD between African and white Americans [16]. Similarly, stroke incidence among Asian populations is much higher than in those of North European descent. Several studies investigating gender as a risk factor have found that there is no substantial difference between the affected male and female population [16]. However, the stroke occurrence rate is 1.25 times more in the male population. In a study the male population was found to be predominant over the female population for the age group ranging from 21–78 years (51% and 49%, respectively) [20].

#### 2.1.2. Modifiable Risk Factors

- High BP

High BP is found to be a powerful precursor for ischemic and ICH (intracranial haemorrhage). BP > 140/90 mmHg has been reported in 77% population during their first stroke attack. Diabetic stroke patients with BP < 120/85 have 50% less lifetime risk than the stroke patients with hypertension (high BP) [21]. More than 50% of the lacunar stroke patients have been found to suffer from hypertension (Baseline data by SPS3) [22].

- Cardiac diseases

Atrial fibrillation (AF) is generally undetectable but clinically treatable. By using outpatient telemetry in cryptogenic stroke/TIA patients for 21–30 days, the atrial fibrillation rate of 12–23% has been detected. With each passing decade, the AF incidence almost doubles above 55 years of age [23]. Almost 50% of cardioembolic strokes are due to AF. The risk of stroke due to AF increased from 1.5% for the age group 50–59 years to 23.5% for 80–89 years [24]. Above the age of 80 years, it has been seen that one out of four stroke cases were due to AF [25,26].

- Smoking

Smoking doubles the risk of stroke. In the Nurses’ Health study and Framingham study, it was revealed that smoking reduces the stroke significant risk within 2–4 years. This positive stroke reduction trend was observed among moderate and heavy smokers of all age groups [23].

- Diabetes Mellitus (DM)

Diabetic patients are more likely to have atherogenic (hypertension, irregular blood lipids, and obesity) risk factors, and such patients have more susceptibility towards atherosclerosis (blocked arteries) [26]. DM has been confirmed as an independent risk factor for causing ischemic stroke through various control and epidemiological studies [27]. In a study by Honolulu Heart Program, it has been revealed that the population suffering from DM has twice the risk of experiencing a thromboembolic stroke, and glucose-intolerant people have double the risk of brain infarction than non-diabetic people (by Framingham) [28]. Besides DM, Hyperinsulinemia is also considered the risk factor for ischemic stroke. In a study by Smith, Ebrahim in 2005, DM was found as the risk factor for intracranial stenosis, which triggers the formation of atherosclerosis. Moreover, in an autopsy study by Hon Kong, the impact of diabetes over stroke was analyzed, leading to the conclusion that DM was a toughened risk factor for having intracranial stenosis [28,29].

- Alcohol consumption

Stroke studies have shown an ischemic stroke in curvilinear relation with low alcohol consumption having a protective effect and boosted risk with excess consumption [23]. Women were at higher risk than men with 3 drinks/day [22]. Heavy alcohol consumption may be the risk of any of the reported stroke types. The risk of small artery occlusion is associated with high alcohol intake in ischemic stroke.

- Obesity

Abdominal obesity is an independent risk factor of stroke in all ethnic groups. This is because obesity is a crucial contributor to increased hypertension and coronary heart diseases. Thus, there should be an emphasis on weight reduction and obesity prevention in every stroke prevention and rehabilitation program [30,31].

- Hyperlipidemia

Hyperlipidemia is directly associated with coronary heart disease, but uncertainty exists in describing its relationship with stroke [32]. The protective influence of high-density lipoproteins (HDL-cholesterol) over atherosclerosis has been reported in various studies. Intracranial stenosis is directly linked with dyslipidemia, especially with high cholesterol [33]. Synergic effect exists between lipoproteins, DM, and intracranial occlusive disorder. Increased levels of LDL (low density lipoproteins) has also been found a risk factor for causing intracranial stenosis [34]. However, an impenetrable correspondence between plasma lipo-concentration and stroke risk has not been established [35]. Low HDL concentration <0.90 mmol/L, high triglyceride level > 2.30 mmol/L, and hypertension have been associated twofold and thus impact the risk of stroke morbidity. Lipids play a prevalent role in stroke risk [36].

## 3. Restoration of UL Mobility

Recovery of patients is a priority as it enables these patients to perform activities of daily living independently, reducing the demand of caretakers and healthcare personnel and providing an opportunity for these patients to resume social participation, thus increasing their quality-of-life [37]. Although restoring UL function in post-stroke patients is essential, regaining the full motor function may not always be achievable, depending on the extent of disability [38]. In a stroke population with some residual muscle activity, exercise or physical therapy dependent brain plasticity has been found to restore the sensory-motor function [39]. Several pilot studies have shown promisingly positive results of robot-assisted rehabilitation for recovery and plasticity following a stroke [40]. Assistive technologies (prosthetic limbs and devices) are a viable option to replace the human body’s lost function [41]). These technologies can move the disabled hand or electrically stimulate the muscle to create muscle contractions in limbs. Paralyzed or atrophied muscles can be restored with neuromuscular electrical stimulation (NMES) using long term implanted system or surface electrodes [42]. Patients suffering from stroke or lower SCI or C1–C4 injury sometimes completely lose the muscle motor action due to motor neuron damage making the restricted use of NMES [43]. Multidisciplinary and supportive services are required to rehabilitate hemiplegic patients in stable condition beginning 48 h after the disease onset [44]. Generally, inpatient and outpatient services are beneficial for both patients and their families, but in a larger perspective, these services have their practice standards regarding diagnosis, prevention, treatment, and rehabilitation, which can vary [44]. Speech, occupational, and physical therapies play an important role in improving the patients’ skills (motor, verbal, etc.), helping create such an environment where the patients with minimum interference of attendants can function. Further, the well-structured and autonomous physical rehabilitation systems help minimise all the infrastructural, mobility and accessibility barriers with orthotics, prosthetics, and electrics devices such as wheelchairs and walkers [45]. Adaptive plasticity has been shown to play a crucial role in motor recovery after stroke for a long time. However, there has always been difficulty identifying the precise mechanisms and neural structures. For the last 15 years’ researchers have conducted numerous studies and controlled trials in animal models and actual stroke patients, concluding that there is a space for adaptive plasticity in the brain after injury or stroke [46]. After the injury, there are various functional variations in the cerebral cortex and it undergoes significant structural changes for several weeks or months after injury/stroke onset [46]. Results of the cortical reorganization indicate marked functional variations after stroke in primate models [47]. Moreover, impaired limb repetitive task practices cause a modulatory impact on cortical plasticity. Training therapies can likely influence the reorganizational mechanisms in the cerebral cortex, thus promoting functional recovery [47]. Immediate, multisensory, and intensive rehabilitative interventions in stroke patients effectively regain functional activities on impaired limbs. However, the mechanisms of neurological recovery after stroke are still not well understood [48]. However, there is experimental evidence that intervention of more than one therapeutic technique is helpful for fast motor recovery, and cerebral plasticity undoubtedly plays a central role in rehabilitation of neural pathways. Various specific therapeutic UL exercises influence cerebral plasticity in stroke patients. In a well-designed rehabilitative system, there should be a feedback system, as it helps in achieving faster and better functional outcomes [49].

### Rehabilitation Therapies

The purpose of this literature review is to provide a detailed overview of the stroke’s morbidity, prevalence, risk factors, social burden, and relevant economic aspects in terms of treatment and care services. This section of the review has discussed various technology-enabled techniques Table 5 to rehabilitate the UL and restore its motor activity after stroke or injury.

- Functional Electric Stimulation

In a pilot study [50], the role of Functional Electric Stimulation (FES) in the recovery of UL motor function was tested in the early stages of stroke. Randomized (Open-label block) trials were applied during inpatient treatment and continued at home. Seven patients received FES plus task-oriented therapy, and control group subjects (*n* = 8) received only task-oriented therapies. All the patients could move their arms freely after the therapy session for 12 weeks. To check the improvement in arms’ functionality, box and block (B&B), Modified Fugl-Meyer (mF-M), and Jebsen Taylor light object lift (J-T) tests were recorded, and the results are shown in Table 6.

In a randomized controlled (double-blinded) study [51], the effectiveness of FES-functional electric stimulation with bilateral training activities was observed for the UL rehabilitation sample of 20 subjects recruited six months after stroke onset. They completed 15 sessions of training. Self-triggered mechanisms synchronized with bilateral UL activities (stretching activities = 10 min, occupational therapy = 60 min, and FES with bilateral task = 20 min) were applied on the participants of the FES group, and their motion was detected via accelerometer. In contrast, the control group participants received occupational and stretching therapies with placebo stimulation for the same duration. Functional test for hemiplegic UE (FTHUE), grip, Modified Ashworth scale, Fugal-Meyer test, forward-reaching distance, and active range of motion test were applied with the following outcomes Table 7.

In 18 sessions by [52], five hemiplegic stroke patients received FES therapy to their triceps, finger extensors, and anterior deltoid for one hour. Different functional tasks such as switching, pressing, and closing a drawer were performed with natural objects during each session. Saebo-MAS and Microsoft Kinect were used to collect the kinematic data. Action Research Arm Test and Fugl-Meyer tests were performed to check the pre and post arms’ functionality. Test score significantly improved for FES assisted therapy by 4.4 points. A feasibility study has shown that economical hardware amalgamated with modern FES controllers can substantially improve the UL motor function. Cyclic (1/2 channel stimulator) NMES-neuromuscular electric stimulation has been applied in numerous randomized controlled studies with encouraging results for acute/subacute patients suffering from hemiplegia [53,54,55]. Patients with some residual muscle activity at baseline have shown more satisfactory results with the application of cyclic NMES [56]. Hemiplegic patients with subluxation randomly assigned to experimental long and short duration subgroups for six weeks (6 h/day) received FES-functional electric stimulation therapy at posterior deltoid and supraspinatus muscles in a study by [57]. They reported that the Fugl-Meyer score significantly improved the arm’s motor function. The effectiveness of FES-ET (functional electric stimulation exercise therapy) during the subacute phase of stroke was tested [58] in a comparative controlled random low and high-intensity treatment study. Nineteen UL-Hemiplegic patients (men = 10 and women = 9) of the age 60.5 ± 5.8 years with average disease duration 48 ± 17 days received FES therapy with adequate encouraging outcomes. In the analysis of randomized controlled studies, FES is an effective treatment for patients > 18 years with a stroke duration time of 2 months. However, no significant improvement was observed in studies where FES treatment was initiated after one year of stroke onset [59].

- Non-Invasive brain stimulation-IBS (t-MS and t-DCS)

According to [60], Transcranial Magnetic Stimulation (TMS) and transcranial direct current stimulation (tDCS) satisfactorily affect the cortical excitability and motor function, thus increasing the clinical arena for the beneficial and customarily use of NIBS as the neurorehabilitation treatment for stroke patients. TMS can alter the cortical activity by creating a transient magnetic field and depolarizing the neurons depending upon the field strength, coil shape, frequency, and duration [61,62]. tDCS (transcranial direct current stimulation) (1–2 mA) using two-surface electrodes can depress or increase the regional excitability in the motor cortex through neuronal depolarization [63,64]. According to [65], NIBS (tMS and tDCS) can modulate the cortical excitability with satisfactory long-lasting effects. In placebo-controlled studies, almost 1000 stroke subjects have been included to achieve UL-motor recovery. High frequency > 3 Hz TMS and low frequency < 1 Hz r-TMS were applied to increase and decrease the excitability of the ischemic cortex, respectively; these non-stimuli have shown constructive effects on neurological scales, functional disability score, and treatment duration of the patients [66]. Disturbed neural pathways are remodelled, and networks between the two hemispheres are modulated by applying NIBS (TMS and tDCS) in post-stroke neuro-rehabilitation by [67]. Neurophysiological alterations have been reported in stroke patients by applying the NIBS-TMS technique [67,68]. NIBS can directly increase the excitability of the motor cortex (ipsilesional). Motor recovery and learning can be increased by applying NIBS directly/indirectly [69]. Pairing up the NIBS with motor training instead of alone results in prolonged functional neural plasticity and performance improvement in the ipsilesional motor cortex [70]. These noninvasive brain stimulation techniques have proven beneficial for modulating brain function and plasticity. tDCS and r-TMS are powerful means to regulate cortical excitability, bring alterations in the motor cortex and thus enhance the motor function of UL after stroke. Neuromodulation with NIBS is a safe, effective, and feasible method to assist recovery from motor impairment after stroke or injury, and no side effects (seizures) have been reported so far [71,72,73,74]. A total of 523 stroke patients were included in a review of 23 studies, including variables such as post-stroke duration, the number of tDCS sessions used, methodology, and type of motor therapy performed. Positive results have been recorded in patients with chronic stroke with the application of anodal tDCS for the motor recovery of UL. No data has been found for subacute stroke. The effect of anodal tDCS is still unclear for the recovery of hand function in the subacute phase of stroke by [74].

- Epidural cortical stimulation (Invasive)

In a single-blinded, prospective, and multicentre study, the safety, efficacy, and feasibility of Epidural Electrical Motor Cortex stimulation (EECS) were assessed for improving the motor function of UL in the ischemic stroke patients suffering from moderate to severe hemiparesis. A total of 104 patients in the experimental group and 60 patients in the control group were included within four months of stroke onset. Epidural six-contact lead was implanted for EECS in the patients of the experimental group perpendicular to the primary motor cortex and pulse generator. Both the groups received rehabilitation therapy for six weeks. Arm motor activity was tested using the arm motor ability test (AMAT), Fugl-Meyer scale, and follow-up assessments. Post hoc comparison tests were performed to determine the treatment effect difference between the control and experimental groups. Test results showed a better recovery rate among the patients of the experimental group who received the EECS therapy compared to the control group [75,76]. For the rehabilitation of UL-upper limb’s motor activity, electric stimulation of the cortex is emerging as a reliable approach. It has been observed from different animal models that cortical stimulation and motor learning facilitate cortical remodelling and long-term potentiation by altering the intracortical inhibitory circuits [76]. Neurological characteristics of the patients suffering from poststroke pain were identified, indicating the satisfactory response to MC-motor cortex stimulation [77]. In this study, 31 patients were treated through the stimulation of the motor cortex; 15 (48%) patients showed an excellent response (>60% reduction in pain). In 13 patients, satisfactory/good pain control was achieved after stroke onset. There was no significant relationship observed between the sensory symptoms such as hypesthesia, dysesthesia, allodynia, hyperpathia, and pain control. However, it has been concluded that the pre-operative evaluation of the motor weakness in painful areas is beneficial for predicting the favourable response towards motor cortex stimulation in controlling the post-stroke pain [77]. Intractable pains have been treated using DBS-deep brain stimulation for more than 50 years. A meta-analysis has been performed to understand better the role of deep brain stimulation in relieving post-stroke pain. Inclusion criteria were based on the clarity of protocol and patient characteristics. The stimulation trial was successful in 50% of patients suffering from post-stroke pain and 58% of patients with permanently implanted treatment for ongoing pain relief [78]. DBS-deep brain stimulation is an effective and remarkably safe treatment for movement disorders. It is being applied to various psychiatric and neurologic disorders, including refractory epilepsy. A review examined the use of deep brain stimulation for the treatment of epilepsy using the known targets and mechanisms of seizure control and neuro modulation [79]. Although deep neuromodulation for the treatment of epilepsy has a very long experimental history, precise stereotactic techniques and epileptogenic networks are now better understood. Robust trial designs are combined to improve the quality of evidence and make the DBS a feasible, trustworthy, and viable treatment option. However, the underlying mechanisms, stimulation parameters, and anatomical targets are still the areas of active investigation [80,81,82].

- Post-Stroke VR rehabilitation

Interactive video games and virtual reality have emerged as viable treatment approaches for stroke rehabilitation. Commercial gaming is rapidly being adopted in clinical settings for cognitive, speech and physical rehabilitation [83]. In a case study the efficacy and reliability of virtual reality for UL activity and function were observed [83].Twenty-nine stroke patients (women = 17, men = 12) aged 43–85 years were included in three different studies to investigate the effect of VR technology for the rehabilitation of stroke. All the included stroke subjects responded positively towards VR activity station. A considerable change in attitude was observed when stroke subjects were exposed to VR computer games [84]. 

Besides functional activity, other factors such as balance, gait, cognitive function, global motor function, quality of life, and participation restriction were also examined. Results indicated that VR effectively improves UL function and daily life activities when used as a complement to usual care. Databases from different sources (Medline, AMED, Proquest, CINAHL, and Psych-Info) were collected [84] to assess the utility of VR for stroke rehabilitation. A total of 11 studies met the inclusion criteria (5 = UL rehabilitation, 3 = gait and balance, 2 = cognitive interventions, and 1 = both UL and lower limb rehabilitation). VR was observed to be a safe and potentially exciting tool for stroke rehabilitation, but the evidence base has been found too limited by power and design issues to permit the definite assessment of its value [85,86]. Although the findings of this review are positive, the evidence level is still weak-moderate in terms of research quality.

Further controlled studies are warranted. Between 50 and 75% of stroke patients suffer from persistent motor impairment of affected UL. Hence, better training strategies for motor training functions need to be identified. Although virtual reality is emerging as a practical treatment approach, its effectiveness needs to be established appropriately. Immersive VR vs. no therapy in UL rehabilitation secured level-1b evidence, level-5 evidence for VR therapy vs. conventional therapy, level-4 evidence showed conflicting results for non-immersive VR vs. no therapy, and level-2b evidence for non-immersive VR therapy vs. conventional therapy [86]. Current evidence of using VR for UL rehabilitation is limited in effectiveness, but it is sufficiently encouraging for justifying additional trials in the stroke population. A PC-based virtual reality system was developed to rehabilitate hand motor activity in stroke patients.

For these two I/P devices, Rutgers Master-RMII and Cyber-Glove were used to allow the patient to interact with the virtual environment. Different target levels based on performance were designed for increasing the patient’s motivation and individualizing the exercise difficulty. Pilot trials in the clinical environment were performed. The working protocol was applied on chronic patients for two weeks regularly. Experimental outcomes indicated a satisfactory improvement in hand parameters. Subjective evaluation was positive, indicating VR as a practical approach for stroke rehabilitation [87,88]. Virtual reality is emerging as well accepted stroke rehabilitation therapy. A review assessed the safety, efficiency, and feasibility of VR therapy for the rehabilitation of UL motor activity. Thirty-seven randomized controlled studies with 1019 stroke subjects were included in this review. VR was a more effective and satisfactory treatment than conventional therapy for improving the UL functional activity [89,90]. VR environment is now considered a promising therapeutic approach for ADL rehabilitation after stroke, especially in the subacute phase. VR tools will have the potential to be used in homes, providing additional therapeutic practice besides formal therapy sessions. VR will likely be an important contributing factor in rehabilitation services in the near future. VR urges therapists to engage with the gaming groups and engineering applications to explore the innovative approaches for delivering viable rehabilitation programs [91,92,93].

- Task-oriented muscle therapy

Functional safety and impairment efficacies of a task-oriented approach have been evaluated for the patients suffering from post-stroke UL disability. Twenty subjects were recruited in a single-blinded randomized cross-over study. Subjects were randomly divided into two groups (*n* = 10 immediate) and (*n* = 10 delayed intervention). The first group received task-oriented therapy for six weeks (3 h/week) followed by no-intervention control for six more weeks. However, there was a reverse order followed for the second group. Canadian Occupational Performance Measure (COPM) functional measures, MAL, and WMFT (Wolf motor function test) was used to measure the motor activity of UL. The score of functional change was higher for the task-oriented (TO) group. A TO approach appears to be a viable and effective post-stroke UL rehabilitation technique with considerable clinical functional improvement [94]. In a review, various databases (Medline, Embase, Cochrane, and CINAHL) were searched to identify the studies related to the TO approach for post-stroke rehabilitation. To ensure the quality, only randomized controlled trials were included. They concluded that TO training for stroke patients is a reliable and safe way to enhance the functional outcomes and overall quality of life [95,96]. Repetitive task training-RTT is an active practice approach to enhance motor activities in stroke patients. Cochrane, MEDLINE, Embase, AMED, and CINAHL randomized controlled group trials were assessed to determine repetitive task training-RTT effectiveness for UL. Low-quality evidence showed that repetitive task training enhances arm functionality (participants analysed = 749). Moderate quality evidence was recorded that RTT improves walking distance (Participants analysed = 610). Significant differences were found between the groups of upper and lower limb functionality. Intervention type, time, and dosage did not modify the effects. However, there was insufficient evidence to be sure about the adverse events [97,98]. For optimising locomotor relearning in stroke patients (chronic), a treadmill was used as a task-oriented-TO training/exercise paradigm. There were beneficial effects observed in the training session of 6 months. Cardiometabolic fitness, ADL task performance, leg strength, and energy cost for hemiparetic gait were significantly enhanced [99,100,101].

- Robot-assisted therapy

Post-stroke rehabilitation is progressing towards deriving more integrated therapeutic muscle training approaches to avoid long-term muscle impairments. In this regard, robot-assisted stroke rehabilitation has shown some promising results. Functional activities of robot-assisted therapy have been extensively examined, giving positive but unsatisfactory results during clinical trials [102]. To address some of the limitations noted, state-of-the-art robot-assisted therapeutic systems have been proposed to rehabilitate the upper and lower limb after stroke [102]. Robot-assisted muscle training devices are being used for post-stroke rehabilitation and to help improve arm functionalit [103]. The effectiveness of robot-assisted and electromechanical arm training to improve daily living activities and muscle strength has been assessed.

Moreover, safety, feasibility, and acceptability factors have also been examined. Randomized controlled studies comparing the robot-assisted and electromechanical therapies were included along with placebo interventions/no-interventions after stroke. A total of 45 trials (participants = 1619) were included in the study. Participants who received both pieces of training after stroke onset improved their daily life activities, muscle strength and arm function. High-quality evidence was achieved due to the variations in duration, intensity, treatment and measurement used [104]. Loss of UL functionality is a common consequence of stroke. Robot-assisted therapy may improve the muscle activity of the arm. The effectiveness of EULT-enhanced UL therapy and robot-assisted therapy using MIT robotic gym has been compared based on usual care and repetitive functional practice. There was no significant improvement in UL motor activity observed in the patients with severe functional disability. The result of the study did not support robotic therapy in routine clinical practice [105,106,107].

- Tele rehabilitation

The effectiveness of tele rehabilitation (TR) was assessed for outpatient therapy to improve poststroke residual activities such as motor function, disability, and speech. A comprehensive literature survey was conducted for three different databases. Complete studies addressing TR training sessions were reviewed. Thirty-four articles with 1025 stroke subjects were included in [108]. Different types of TR were reported to be used in related studies, including VR therapy, speech therapy, robot assisted therapy, different motor training sessions, and community-based therapeutic sessions, which revealed that TR is less expensive and equally effective than clinic-based therapy practices. However, TR can be integrated with other therapies such as speech practice, VR, or robotic to achieve more satisfactory results [109]. To examine whether telerehabilitation helps improve the functional ability of stroke patients to perform daily life activities compared to in-person (face to face therapy session) and usual care/no rehabilitation, a systematic review has been conducted (Cochrane group trials 2019, Cochrane library 2019, Cochrane controlled trials, MEDLINE, Embase, and eight additional databases).

Only randomized controlled trials were included in the study. The authors reviewed articles based on prespecified criteria and assessed the risk factors. GRADE was used to assess the viability and quality of evidence. A total of 1937 stroke subjects were included in 22 different trials. Comparisons greatly varied throughout the studies. However, no adverse events were reported related to TR. This is still an emerging field, and more definitive results are required. Results of the studies in which mixed evaluation methods were used are valuable [109,110]. Although activity-based therapeutic sessions for the rehabilitation of stroke patients are very significant in improving functional capabilities and quality of life, some patients, due to various reasons (transportation difficulties, low compliance, and poor access, etc.), cannot receive these services. TR can play an essential role in resolving these issues by providing quality muscle training programs. The feasibility of TR has been assessed for an expanded telerehabilitation program. Thirteen stroke patients received home-based TR under the supervision of an expert physiotherapist. The resulting outcomes were evaluated using different tests (Modified Rankin score, Fugl-Meyer test). A home-based TR system provides a holistic approach for rehabilitation and stroke prevention. More work needs to be focused on extracting more reliable, credible, and satisfactory outcomes regarding TR [111,112,113].

## 4. Discussion

This review covers the socio-economic and therapeutic aspects of stroke. Different therapies available for rehabilitation of the upper limb after stroke have been surveyed. Moreover, a brief statistical analysis regarding disease-related expenditures, i.e., treatments, medication, and care services, is included. Stroke rehabilitation is one of the top areas for researchers and clinical experts as, globally, this disease has a high prevalence rate and economic cost to society. Lifetime care services may be required for a patient, depending upon the severity of the disease. The U.S. average cost for home care services for stroke patients from 2001 to 2005 was registered to be USD 11,145/patient. The risk of stroke is high in the older/elderly population (above 60) as ageing is one of the factors causing a stroke. However, a high surge in stroke numbers is expected in the coming decades if it keeps increasing at a constant rate. Patients suffering from stroke cannot live an independent life as they may need health and care services all the time.

Keeping these facts in mind, it is necessary to address the knowledge gap to formulate effective public health care and rehabilitation policy. More than 150 randomized controlled studies, pilot experimental trials, and meta-analyses were identified from Google Scholar. However, only 114 studies were selected addressing the criteria mentioned above, i.e., stroke statistics and upper limb rehabilitation, mainly in the acute or chronic phase. Therapies such as functional electric stimulation (FES), non-invasive brain stimulation (NIBS) including t-DCS and t-MS, invasive epidural cortical stimulation, tele rehabilitation, robot-assisted therapy, and cerebral plasticity are commonly used for stroke rehabilitation, especially in more advanced economies. Functional electric stimulation (FES) is a valuable rehabilitation treatment in different pilot studies, randomized controlled trials, and clinical practices. Mostly hemiplegic patients with upper limb disability in early stage or full 2–6 months of the disease onset were applied with FES and in some sessions FES in combination with other therapies (task specified, exercise-based, and occupational therapies). FES was a practical rehabilitation approach when applied alone or in combination with other therapies compared to the conventional way of stroke treatments.

More than 1500 stroke patients were included in different placebo and randomized controlled studies to check the feasibility and effectiveness of NIBS (non-invasive brain stimulation). t-DCS and t-MS have been found to alter cortical excitability and increase the motor function of the impaired limb. Modulation in cortical excitability through the application of NIBS generates long-lasting beneficial results. The application of epidural deep brain stimulation on more than 200 stroke patients in different clinical research studies has been found to generate remarkably encouraging outcomes to treat movement disorders. Various AMAT, Fugal-Meyer, and follow up assessments have also given the results favouring this treatment. Almost 800 patients suffering from UL impairment after stroke in acute and chronic phases were tested with specified task-oriented therapies with satisfactory motor recovery rates in different experimental trials. A total of 4594 stroke subjects have been included in various clinical, experimental, and controlled studies proving the other rehabilitative treatments such as robot-assisted, tele rehab services and cerebral plasticity to regain the lost functional ability.

Moreover, VR has been categorized as a modern rehabilitation approach with positive results. Allowing the patient to interact with a virtual gaming environment has been proven to be a practical approach to rehabilitating impaired limbs. However, a strong evidence base is needed for VR to be adopted widely into clinical practice. In the end, it is worth mentioning that this narrative review does not present any meta analysis, but a clear, informative, and quality background of the stroke disease, eventually emphasizing the significance of rehabilitation therapies while explaining the unbearable economic consequences of the disease coming 2–3 decades.

## 5. Conclusions

A comprehensive review addressing the statistical (economic, prevalence, and risk factors) and therapeutic aspects of stroke have been composed. Some of the best available therapies regarding upper limb rehabilitation have been discussed. Different placebo and randomized controlled studies and their experimental outcomes were included to analyse their efficacies and usability. This review aims to bridge the knowledge gap regarding stroke disease and therapies available for the rehabilitation of post-stroke motor impairment.

## Figures and Tables

**Table 1:** Stroke services monthly cost/patient for the year 2015 [5].

Country	Per Patient Cost/Month in USD	Cost/Month in USD per Outpatient Only
Australia	752	Not available
Canada	1444	Not available
Cuba	Not available	616
Denmark	3022	Not available
France	1125	Not available
Germany	996	559
Italy	833	Not available
Malaysia	Not available	192
Netherland	2016	Not available
Norway	2147	Not available
Sweden	768	389
Switzerland	1505	Not available
UK	868	883
USA	4850	773
Multicentric	2385	Not available

**Table 2:** Risk factors for stroke (modifiable and non-modifiable according to the different research studies in Pakistan, Brazil, India, and South East Asia) [11,12,13,14,15].

Modifiable Risk Factors	Non-Modifiable Risk Factors
Hypertension **(65.8%)**	Older age > 65 years
Transient ischemic attack (TIA) **(24.9%)**	Family stroke history
Cardiac Diseases **(29.1%)**	Higher in males
Carotid artery stenosis	Ethnic factor
Atrial fibrillation	---
Hyperlipidemia **(25.5%)**	---
Physical inactivity	---
Smoking **(43.0%)**	---
Diabetes **(41.3%)**	---
Excess alcohol intake	---

**Table 3:** Data were taken from different studies conducted in different countries to assess the prevalence of the risk factors of stroke [12,13,14,16,17,18,19].

(**a**) Prevalence Frequency of Different Risk Factors for Ischemic Stroke in a Study Population in Pakistan (Study included 55 subjects to analyze the prevalence of modifiable and non-modifiable risk factors				
**Risk Factor**		**Male (*n* = 43)**	**Female (*n* = 12)**	**Total (*n* = 55)**
Smoking		32 (74.3%)	0	32 (58.1%)
Familystroke history		22 (51%)	6 (50%)	28 (50.8%)
Dyslipidemia		15 (34.5%)	3 (25.1%)	18 (32.5%)
Obesity		9 (20.8%)	11(91.2%)	20 (17.9%)
Cardiac disease		4 (9.3%)	1 (8.3%)	5 (9.2%)
Diabetes mellitus		17 (38.8%)	3 (24.9%)	20 (35%)
Epilepsy		7 (15.9%)	2 (16.5%)	9 (15.9%)
(**b**) Yearly Awareness, Control, and Treatment Ratio for Stroke Risk Factors in China, Japan, and Taiwan				
**Risk Factor**	**Category**	**China**	**Japan**	**Taiwan**
Hypertension	Awareness	44.7 in 2000–2001, 24 in 2002, and 45 in 2007–2008	54 in 2000 and 66 in males and 73 in females in 2000–2001	22.5 in males and 39.3 in females in 1993–1996
	Treatment	28 in 2000–2001, 20 in 2002, and 36.2 in 2007–2008	46.1 in 2000, 16.4 in males and 33–57 in females in 2000–2001, and 54.4 in 2008	13.4 in males and 28 in females in 1993–1996, 44 in males and 59 in females in 2002
	Control	8.1 in 2000–2001, 5 in 2002, and 11 in 2007–2008	23.4 in males and 28 in females in 2000, 27 in 2008, and 25 in 2009,	2–2.3 in males and 5.1 in females in 1993–1996, 21 in males and 28 in females in 2002
High cholesterol	Awareness	24.4 in 2003–2013	56 in males and 59 in females in 2000–2001	---
	Treatment	9 in 2003–2013	52 in males and 53 in females in 2000–2001	---
	Control	4.2 in 2003–2013	72 in 2009	65 in 2002–2003 in 2006–2007
Diabetes	Awareness	24 in 2000–2001 and 30 in 2010	---	70 in males and 63 in females in 1993–1996
	Treatment	20 in 2000–2001 and 26 in 2010	---	---
	Control	8.4 in 2000–2001 and 39.7 treated patients in 2010	34 from 2000–2002 and 36 from 2006–2008	27.00 in 1998 and 11.2 in 2006 (among patients having insulin therapy)
(**c**) Prevalence assessment of stroke risk factors in a study with 688 patients in Brazil				
**Patients** ***n* = 688**		**Microangiopathy**	**Macroangiopathy** ***n* = 127 (18.5%)**	**Cardio Embolism** ***n* = 195 (28.3%)**
Women *n* = 360 (52.3%)		49.6%	52.3%	53.3%
Men *n* = 328 (47.7%)		50.4%	47.5%	46.7%
Age above 65		72.4%	63.2%	56.8%
Smoking *n* = 164		29.1%	30%	16.9%
Hypertension *n* = 517 (almost in all groups)		92.1%	80.7%	69.7%
Dyslipidemia *n* = 324		50.4%	57.8%	40%
Diabetes *n* = 146		27.6%	26.9%	18.5

**Table 4:** First and second year cost analysis per patient according to modified Rankin scale score for (**a**) Hemorrhagic stroke (**b**) Ischemic stroke [11].

**(a)**		**Inpatient Stay Care**		**Outpatient** **Specialty Care**		**Outpatient** **Primary Care**		**Home Care Services**		**Particular** **Housing Days**	
First year	Month 1–3	No. of Days	Amout in Euros	No. of Visits	Amout in Euros	No. of Visits	Amout in Euros	No. of Hours	Amout in Euros	No. of Days	Amout in Euros
	2	23	20,015	11	3123	13	1525	19	876	2	330
	3	37	31,668	10	2812	11	1310	235	10,904	34	6290
	4	49	42,295	12	3358	12	1374	510	23,684	82	15,071
	5	64	55,370	6	1605	9	1069	501	23,264	170	31,476
	Deaths	14	12,397	1	327	1	127	47	2177	26	4780
	Survivals	39	33,521	10	2898	12	1369	213	9873	51	9494
	Patients	29	25,306	7	1898	7	886	146	6769	41	7661
Second year	Month 12										
	2	2	9784	4	1033	5	626	25	1155	1	230
	3	6	9770	4	1032	6	667	698	32,420	39	7115
	4	7	9032	3	954	6	674	1419	65,931	67	12,448
	5	3	6217	2	656	5	550	689	31,999	250	46,221
	Deaths	12	7431	3	785	4	527	453	21,056	128	23,570
	Survivals	5	8159	3	862	5	588	429	19,931	52	9581
	Patients	5	8095	3	855	5	582	431	20,021	59	10,811
**(b)**	**mRs Scale Value**	**Inpatient Stay Care**		**Outpatient** **Specialty Care**		**Outpatient** **Primary Care**		**Home Care Services**		**Particular** **Housing**	
First year	Month 1–3	No. of Days	Amout in Euros	No. of Visits	Amout in Euros	No. of Visits	Amout in Euros	No. of Hours	Amout in Euros	No. of Days	Amout in Euros
	2	12	20,015	9	3123	13	1525	13	876	1	330
	3	25	31,668	8	2812	12	1310	243	10,904	34	6290
	4	35	42,295	9	3358	13	1374	547	23,684	75	15,071
	5	41	55,370	5	1605	8	1069	392	23,264	213	31,476
	Deaths	23	12,397	2	327	3	127	100	2177	48	4780
	Survivals	22	33,521	8	2898	12	1369	171	9873	40	9494
	Patients	22	25,306	7	1898	10	886	154	6769	42	7661
Second year	Month 12										
	2	3	1704	3	1033	5	626	26	1155	1	230
	3	6	4263	3	1032	5	667	571	32,420	40	7115
	4	8	4899	3	954	5	674	1325	65,931	78	12,448
	5	4	2465	3	656	5	550	741	31,999	265	46,221
	Deaths	14	8736	3	785	5	527	505	21,056	105	23,570
	Survivals	5	3262	3	862	5	588	373	19,931	44	9581
	Patients	6	3744	3	855	5	582	384	20,021	50	10,811

**Table 5:** Included Stroke Rehabilitation Techniques.

Technique	Focus	Strategy	Comparison with Conventional Therapy	Disability
*FES (functional electric stimulation) [50]*	To study the effect of FES on UL rehabilitation	Open-label block inpatient randomized control study	Fast recovery than task traditional task-oriented physiotherapy	Acute phase of stroke
*FES [51]*	Application of FES with bilateral training on UL	Randomized double-blinded controlled study	Test scores for FES intervention showed better improvement	6 months after stroke onset
*FES therapy [52]*	FES therapy on triceps and anterior deltoid	18 sessions of 60 min. therapy with diff. functional tasks	FES therapeutic intervention improved functionality tests score by 4.5 points	Hemorrhagic stroke
*NMES-neuromuscular electric stimulation [53,54,55,56,57]*	To study the effect of NMES application on hemiplegic patients	Cyclic stimulation in randomized control studies	Satisfactory results have been observed	Acute/subacute phase of stroke but applicable in chronic phase as well
*FES [58]*	For analysing the effect of FES in patients with hemiplegia	Randomly controlled FES session of 6 weeks for 6 h everyday	UL motor functions were significantly improved	Hemiplegia with subluxation
*FES-ET [59]*	Potency check of FES therapy	Comparative controlled strategy	Obtained satisfactory results	Stroke subacute phase (UL hemiplegia)
*NIBS [60,61,62,63]*	To test the results of tDCS and tMS	Modulation of cortical excitability	Effective and feasible	Motor disability due to Stroke
*NIBS [64]*	Application of tDCS for UL rehabilitation	Placebo controlled mechanism	Encouraging outcomes in terms of recovery duration	Post ischemic stroke disability
*NIBS [65,66,67,68,69,70,71]*	Neuromodulation using NIBS	Regulation of cortical excitability with r-tMS	safe and effective	UL disability after stroke
*NIBS [72,73,74]*	Application of anodal non-invasive t-DCS as motor therapy	Meta analysis of 23 studies with >500 patients in total	Positive but not-sufficient outcomes to reach any conclusion.	UL disability due to chronic stroke
*Epidural stimulation invasive [75,76]*	To check the efficacy and feasibility of EECS	Single blinded and multicenter study	Better recovery rate was recorded as compared to the control group	Moderate to severe ischemic stroke patients with UL disability
*Cortical electric stimulation [77,78]*	Rehabilitation of motor activity of UL	Stimulation of motor cortex of animal models	Satisfactory results were observed	Disability of UL
*Stimulation of motor cortex [79,80,81,82]*	To understand the neurological characteristics through motor cortex & deep brain stimulation	stroke subjects were included in the studies	48–50% patients showed positive results	Post stroke pain
*VR rehabilitation [83,84,85]*	To understand the effect of VR for stroke rehabilitation	Stroke patients were included in the study	General experience indicated positive results	Post stroke disability
*VR [86,87,88]*	To analyse the efficacy of virtual rehabilitation	Different databases were examined in a review	Sufficient satisfactory results were observed	Functional disability
*VR [89,90,91,92,93]*	Rehabilitation of motor activity	PC-based VR systems were designed and pilot trials were performed	Satisfactory improvements were observed in hand parameters	Chronic stroke patients
*Task-oriented therapy [94]*	To test the functional and impairment efficacies of task-oriented therapy	20 patients were included in a Single-blinded randomized study	Group who received task-oriented exercises showed better recovery rate	Post stroke UL disability
*Task-oriented therapy [95,96,97,98,99,100,101]*	Optimization of locomotor relearning	Aerobic complex task trainings	Motor abilities of the patients improved after therapy session	Chronic stroke patients
*Robotic therapy [102]*	To design a robot based therapeutic system	Robot based training	Positive but not satisfactory	Functional disability
*Robot assisted therapy [103,104,105,106,107]*	To compare the results of EULT and robotic therapy based on MIT robotic gym	Repetitive functional therapy	Not significant improvement was observed in UL functionality	UL disability
*Tele rehabilitation [108,109]*	To check the feasibility of tele rehabilitation system	Outpatient therapy	As effective as clinical based therapies	Motor disability
*Tele rehabilitation* *[110]*	To examine the efficacy of tele rehabilitation	Different data bases from MADLINE, Cochrane, and Embase were collected and analyzed	No adverse events were reported, considered to be an emerging field however more trials are needed	Post stroke motor disability
*Tele rehabilitation* *[111,112,113]*	Use of tele rehabilitation for accommodating the stroke patients on large scale	Activity based therapies	Appears to be a holistic approach	Patients of functional disability

**Table 6:** Improvement Analysis of UL [44].

Test Type	Baseline Score	12th Week Score
1. B&B		
FES group	7.00 ± 0.00	48.00 ± 28.00
Control Group	4.00 ± 0.50	25.5 ± 15.0
2. mF-M		
FES group	23.0 ± 17	51.0 ± 44.0
Control Group	20.5 ± 15.5	39.0 ± 33.25
3. J-T		
FES group	60.0 ± 18.0	5.70 ± 4.20
Control Group	60.0 ± 39.75	10.0 ± 7.87

FES group showed a better recovery rate than the control group (task-oriented only).

**Table 7:** Improvement analysis of UL [50].

Test Type	FES Group Mean Score		Control Group Mean Score	
	Pretraining	Post-Training	Pretraining	Post-Training
Fugl-Meyer test	18.1 ± 7.8	25.8 ± 8.7	19.9 ± 10.00	22.0 ± 9.8
Forward reaching (cm)	12.6 ± 7.6	20.4 ± 9.7	7.7 ± 9.7	11.9 ± 12.4
Grip power (kg)	1.20 ± 1.9	2.20 ± 2.0	1.1 ± 1.59	2.00 ± 2.1
FTHUE	2.5 ± 0.8	3.7 ± 0.5	2.8 ± 0.6	3.1 ± 0.6
Functional independence	76.7 ± 12.0	80.2 ± 6.9	77.3 ± 12.0	77.6 ± 12.0

FES with bilateral UL therapy is better in improving the motor function.

## Data Availability

Data presented in the paper is extracted from published work. The authors of the current study do not have the raw data.
